# Extremophilic Microalgae *Galdieria* Gen. for Urban Wastewater Treatment: Current State, the Case of “POWER” System, and Future Prospects

**DOI:** 10.3390/plants10112343

**Published:** 2021-10-29

**Authors:** Maria Rosa di Cicco, Manuela Iovinella, Maria Palmieri, Carmine Lubritto, Claudia Ciniglia

**Affiliations:** 1 Department of Environmental, Biological and Pharmaceutical Sciences and Technologies, University of Campania “Luigi Vanvitelli”, Via Vivaldi 43, 81100 Caserta, Italy; mariarosa.dicicco@unicampania.it (M.R.d.C.); maria.palmieri@unicampania.it (M.P.); carmine.lubritto@unicampania.it (C.L.); claudia.ciniglia@unicampania.it (C.C.); 2 Department of Biology, University of York, Wentworth Way, York YO10 5DD, UK; 3 INFN—Sezione di Napoli, Complesso Universitario di Monte S. Angelo, ed. 6, Via Cintia, 80126 Napoli, Italy

**Keywords:** microalgae cultivation, extremophile, *Galdieria*, *phlegrea*, *sulphuraria*, photobioreactor, pollutants removal, urban wastewater

## Abstract

Over the past decades, wastewater research has increasingly focused on the use of microalgae as a tool to remove contaminants, entrapping nutrients, and whose biomass could provide both material and energy resources. This review covers the advances in the emerging research on the use in wastewater sector of thermoacidophilic, low-lipid microalgae of the genus *Galdieria*, which exhibit high content of protein, reserve carbohydrates, and other potentially extractable high-value compounds. The natural tolerance of *Galdieria* for high toxic environments and hot climates recently made it a key player in a single-step process for municipal wastewater treatment, biomass cultivation and production of energetic compounds using hydrothermal liquefaction. In this system developed in New Mexico, *Galdieria* proved to be a highly performing organism, able to restore the composition of the effluent to the standards required by the current legislation for the discharge of treated wastewater. Future research efforts should focus on the implementation, in the context of wastewater treatment, of more energetically efficient cultivation systems, potentially capable of generating water with increasingly higher purity levels.

## 1. Introduction

In recent years, the attention of scientific community and world governments has been shifting towards the promotion of technologies for the recovery of material and energy resources from waste streams [1]. The wastewater sector is currently one of the most critical discharge basins of resources for which there is a priority interest in their recovery [2]. First, attention is on the energy, whereby the wastewater sector is currently responsible for about 1% of the worldwide electricity consumption, which is still generated by fossil fuels in most world areas [3,4,5]. Together with energy consumption, also important is the large amount of nutrients and organic substances enriching municipal wastewater, which is not possible to effectively recover with the purification systems currently most widespread worldwide, such as activated sludge processes [6,7]. Indeed, urban wastewater is very rich in macro- and micronutrients, of which an outstanding example is phosphorus [8]. Around phosphorous there is particular attention because, while it represents such a fundamental element for all living forms (e.g., formation of cell walls or metabolic reactions for energy release), it is nevertheless one of the least available elements in nature and, simultaneously, one of the most required from the fertilizers industry [9]. van Dijk et al. [10] attributed to wastewater the responsibility of more than 50% of the phosphorus flux out of the European Union macrosystem.

In adopting circular economy policies in the wastewater sector, Smol et al. [11] proposed six actions, mirroring the well-known waste management hierarchy: reduction, reclamation, reuse, recycling, recovery, rethinking. These six actions addressed the recovery of material and energy resources from wastewater, the development of effective strategies for the reuse of potable and non-potable wastewater fractions, the implementation of sustainable processes in which emissions and waste fractions were minimised as much as possible.

Against this background, introducing the “microalgae” element into the wastewater sector represented one of the most promising breakthroughs in recent decades [12,13]. The concept behind the process was that urban wastewater could be used as a growth medium for the cultivation of biomass with high commercial value, and that these organisms could sustainably contribute to the purification of the effluent, during the growing process [14,15]. In this way, it would be possible to perform both a bio-cleaning action of the sewage and a bio-recovery of nutrients, which would be returned to the trophic chain through these organisms. This process, as outlined by Perin et al. [9], would help to mitigate the negative environmental impact of practices strongly relying on a continuous macronutrients supply, such as agricultural processes. The use of microalgae to recover nutrients from waste streams also potentially addresses the first point of the waste management hierarchy proposed by Smol et al. [11], contributing to a more efficient use of finite resources that are increasingly difficult to extract.

This approach was initially envisioned by Oswald et al. [16] in the middle of the last century, but only recently it received a significant boost in implementation, driven by increasingly stringent sustainability policies. The so-called biorefineries (as we currently refer to systems for the sustainable production of algal biomass used to generate byproducts and/or energy) are indeed complex systems from the operational point of view, for which the running costs very often exceed those of conventional wastewater treatment plants (hereby WWTPs) [12,17,18]. To promote the adoption of algal-based systems with acceptable payback times, it should be necessary to use microorganisms which could be profitable in terms of how to reuse the biomass, for example: (i) through the extraction of biocompounds with high added value, for which there is a large market [19]; (ii) for the production of fertilizers, to reduce the amounts of fresh nutrients hardly extracted or mined [9,20,21]; (iii) for the sustainable biorecovery of heavy or precious metals and Rare Earths Elements, through processes of bioaccumulation and biosorption [22,23,24].

For this reason, to the present day the relevant literature abounded in studies that focused on those strains with high content of pigments, starches and especially lipids, the latter being used to obtain biofuels through transesterification processes [25]. Algal species, however, tend to have different physiological requirements, related to process temperature, pH of the cultivation medium, presence/absence of heavy metals and potentially toxic elements in the effluent, presence/absence of light radiation in the cultivation system, as well as the ability to perform autotrophic, heterotrophic or mixotrophic metabolism. In designing an effective bioreactor for the cultivation of these organisms, the setting of these parameters must be carefully programmed, while also being aware that the physiological optimum of a microalgal species could be similar to that of the majority of the bacterial community naturally present in wastewater [26]. In such a case, an issue of concern could be the possibility of interspecific competition for the resources available, as well as potential attack by invading species and pathogens (especially in systems exposed to open air) [27]. To overcome this risk, using microalgal specialists such as those adapted to extremophilic environmental conditions, and which require those conditions for their metabolic activity, could potentially enable a safer microalgae cultivation [21,28].

Microalgae belonging to the genus *Galdieria* (class *Cyanidiophyceae*) are thermoacidophilic extremophiles, usually adapted to thrive as algal mats in volcanic areas, sulphuric environments, and acidic hot springs [29]. Since adaptation to such extreme environments is part of their nature, these organisms are able to tolerate the presence in the surrounding environment of heavy metals and substances known to be toxic to the majority of other organisms [30,31]. Their ideal physiological conditions also include acidic pH conditions down to 0 and temperatures up to 56 °C, for which they developed a thermo-tolerant cell wall [32]. The extractable phycocyanin, of which they are great producers, was found to be also much more stable in relation to temperature [33]. Another metabolite of high commercial interest that could be extracted from *Galdieria* is a highly branched, low molecular weight and low temperature soluble glycogen, which is highly attractive to the food and nutraceutical industry [34]. But *Galdieria* is also an extraordinarily capable organism for removing heavy and precious metals from polluted waste fluxes, e.g., through biosorption, and it is therefore well suitable for bioreclamation applications in the e-waste sector [30].

The microalgae of the *Galdieria* genus attracted a lot of attention in recent years because of their appealing growth conditions and versatile applications [21]. Producing *Galdieria* from a cheap, sustainable, and nutrient-rich growth medium such as municipal wastewater is an ambitious goal, towards which research only recently began to move [13,35]. Therefore, this review aimed to analyse the current state of the art on the use of microalgae of the genus *Galdieria* in contact with municipal wastewater, for the simultaneous cultivation of biomass and removal of contaminants, and to outline prospects for experimentation.

## 2. Screening Criteria for Scientific Articles

To realise this review, the guidelines of the PRISMA statement were followed [36].

In the first identification phase, publications were collected using Scopus scientific database (https://www.scopus.com/search/form.uri?display=advanced, last access on 29 September 2021). Scopus was selected as the reference database because it provided a higher number of documents, according to the keywords used for searching and the eligibility criteria. The search was done using as search fields the following sections: article title, abstract and keywords. Since municipal wastewater is usually referred to as “sewage”, “waste water” and “wastewater”, the following 3 pertaining combinations were initially used for the search, whose results as number of documents are reported in brackets: “*Galdieria*” and “*sewage*” (#4 documents); “*Galdieria*” and “*waste water*” (#16 documents); “*Galdieria*” and “*wastewater*” (#36 documents). Since the last combination returned the highest number of publications, it was decided to use it as a reference for the research.

As a first screening operation on the 36 documents, all publications that were not scientific articles type (e.g., reviews, conference papers) were excluded. The final Scopus query string for the search was as follows: “TITLE-ABS-KEY (*Galdieria* wastewater) AND (LIMIT-TO (DOCTYPE, “ar”))”.

Subsequently, all the resulting articles (#31) were read and organized in relation to: (i) microalgal strain used in the study, (ii) type of effluent used in the experiments, and (iii) objectives of the research (Table 1).

Among the 31 scientific articles from 2014 to 2021, the documents that did not address any practical application of municipal wastewater (#10) were excluded from the study because they did not meet the aims of this review.

## 3. Current State of the Art

### 3.1. General Framework

Using the criteria applied at the screening stage, a total number of 21 articles were identified as suitable for study and discussion in the present review, the first article being published in 2014 [57]. Except for a single experimentation involving the species *Galdieria phlegrea* (strain: ACUF 784.3) [46], all remaining studies relate to the implementation of a single-step algal process that mainly involved one single strain of *G. sulphuraria* coupled with urban wastewater for the removal of organic carbon, nitrogen and phosphorus, removal of metal ions and hydrothermal liquefaction for the production of biofuels from the produced biomass. Since the research was performed in Las Cruces (New Mexico) the idea was to implement a system at the local WWTP which could be optimized for hot and arid regions, where high diurnal temperatures were typical and water scarcity was a serious concern. Meanwhile, given that the species *G. phlegrea* proved to be not a strictly thermophilic organism [65], in di Cicco et al. [46] the purpose was to investigate the performance of *G. phlegrea* grown in urban wastewater as an alternative to *G. sulphuraria* in less extreme climates. However, considering that the Las Cruces algal system denominated “POWER” (Photosynthetically Oxygenated Waste-to-Energy Recovery) (first mentioned in [59]) was the most comprehensive example of municipal wastewater treatment with a microalgae of the *Galdieria* genus, it was set as the main benchmark for the present review, while the findings concerning *G. phlegrea* were integrated in the discussion.

### 3.2. Objectives of the Studies—Focus on Las Cruces POWER System

During the 7-year timeframe of the POWER project, many experiments were performed to assess such process (Table 1), and all these experiments could be resumed in the following four main branches:

1. feasibility of using raw wastewater as a growth medium for the selected strain;
2. efficiency of this system for removal of pathogens;
3. hydrothermal liquefaction to maximise energy recovery and biomass production;
4. performance of the system in a simulated continuous process.

Municipal wastewater samples used for all experiments were collected downstream of the primary clarifier at the municipal WWTP in Las Cruces, New Mexico (US) [57]. Experiments were conducted both in a controlled environment (laboratory) and outdoors to mimic conditions as real as possible. The cultivation system for outdoor cultivation was a closed polyethylene bag (1 m wide × 3 m long) into which air enriched with CO_2_ (1–2%) was injected at a pressure ~10% above atmospheric values [57]. The PBRs were transparent and laid in a horizontal position; internally, bags were supported by a PVC skeleton, with an electro-mechanical paddle wheel installed in the centre of each bag providing circulation to the cultures [59]. The operational batch volume was 700 L, starting with 400 L of effluent and 300 L of pre-adapted cultures [13].

The experiments involving *G. phlegrea* were performed only at a laboratory scale, in batch mode, using 1L Erlenmeyer flasks on a linear shaker.

### 3.3. Feasibility of Using Raw Wastewater as a Growth Medium for Galdieria Gen

The first experiments within the context of the POWER system had the purpose of testing whether *G. sulphuraria* could grow in raw wastewater, using as a reference the standard growth medium *Modified Cyanidium Medium* (MCM), used for the cultivation of algal stock. The composition of MCM and the complete description of the experiments was fully described in [13,47,57,58].

Overall, results showed that *G. sulphuraria* could be grown in primary effluent at growth rates higher than what achieved with the baseline MCM. In particular, growth with primary effluent reached a density of 2.7 g L^−1^, while with the standard media it saturated at 1.6 g L^−1^. At the end of the exponential phase, all media reached the same density of 1.2 g L^−1^ [57]. In general, growth in primary effluent showed the highest biomass density at the end of a 10-day experiment [58]. Temperature did not affect the growth [57]. A possible interpretation provided by the authors for the better growth at late-stage was related to the presence of trace-metals into the growth medium [47]. Typically, *Galdieria* developed in hot and volcanic acidic groundwaters, where are usually present high concentrations of metals, including iron, copper, manganese, and zinc [66]. In order to thrive in such habitats and cope with these potentially toxic elements, it is reasonable to think that *Galdieria* could be equipped with high-affinity transport systems for metal ions. Indeed, in 2013 Schonknecht et al. [31] determined the genome sequence of *G. sulphuraria*, in order to explain the extremophilic and metabolically flexible lifestyle of this microorganism. The results of the sequencing showed that *G. sulphuraria* inherited through horizontal gene transfer from archaea and bacteria many of the characteristics that enable the survival in such hostile environments and, in this specific case, the ability to cope successfully with the presence of toxic metals in the surrounding environment [31]. The study of Schonknecht et al. [31] revealed that more than 5% of the genetic code of *G. sulphuraria* encodes for membrane transport proteins and, among these, several membrane transporters were found to be specific for divalent metal cations, allowing the selective uptake of essential or precious metals and paving the way for their recovery from waste materials, as also demonstrated by Ju et al. [22] and Minoda et al. [67]. Considering this aspect, high-purity water and chemicals used in the experiments to prepare the laboratory growth medium might not provide sufficient trace metals, especially when cell densities are higher. On the other hand, municipal wastewater would be expected to have higher levels of physiologically important metals, which might be the reason for the growth stimulation at late stages [47]. Removal efficiencies of ammonium and phosphate over 7 days among all the different test media ranged between 88.3–90.5% and 95.5–98.1%, respectively [57]. After 10 days, both N and P ionic species had a concentration near zero (below the detection limit) [58]. In terms of removal rates, results indicated 4.8 mg L^−1^ d^−1^ of ammonium and 1.21 mg L^−1^ d^−1^ of phosphate removed from wastewater [57]. Overall, biomass yield in primary effluent against removal of nitrogen resulted in 27.4 g_biomass_ g_nitrogen removed_^−1^, which was a value (i) more than 70% higher than the theoretical yield of 15.8 g_biomass_ g_N removed_^−1^ estimated from the canonical empirical molecular formula of Redfield et al. [68], and almost 10% higher than the average yield reported in the literature for other species (25.7 g_biomass_ g_nitrogen removed_^−1^) [57]. Wanting to compare the growth performances in this PBR with the values achieved in open pond cultivation systems with municipal wastewater, final cell densities in POWER system were 3–5-fold higher (e.g., Park et al. [69]). This significant result translated into the possibility of reusing municipal wastewater (instead of freshwater) to cultivate microalgae in arid environments with the advantage of having lower energy costs. Indeed, when harvesting biomass with higher cell densities and lower humidity, the energy required for drying stage decreased [20].

Furthermore, performances of the system concerning biomass production, as well as ammonium and phosphate removal, were evaluated not only against different growth media, but also against different pH conditions (2.5 and 4). Results reported that pH did not impact the process to any extent [47]. In addition, *G. sulphuraria* was able to actively adjust the pH of the growth medium, attaining a stable value of 4 in only 3 days, an ability also documented in other recent studies [70]. These findings about pH have major and direct implications on the feasibility of the algal-based system, as they suggest potential cost reductions for the purchase of pH-modulating chemicals.

Based on this extensive and well-established background, experiments were performed to understand whether the *phlegrea* species could grow and remove contaminants with performance similar to the *G. sulphuraria* strain CCMEE 5587.1 used in the Las Cruces experiments. Here, as mentioned in the previous section, the experiments were batch processes conducted in volumes of less than 1L (full details can be found in [46]). Despite this important difference in terms of cultivation process, the physiological performances shown by *G. phlegrea* were consistent with those reported for *G. sulphuraria*, with biomass growth rates of approximately 23.2 g_biomass_ g_N removed_^−1^, and ammonium and phosphate removal rates of approximately 4.0 and 1.5 mg L^−1^ day^−1^, respectively. It should be noted that in the experiments performed with *G. phlegrea*, the urban wastewater was sampled in a WWTP with a strong dilution problem [71], so that the initial ammonium and phosphate concentrations of the wastewater were much lower than those found in the municipal wastewater from the Las Cruces treatment plant [46,57]. According to the relevant literature, higher values in terms of both biomass production and pollutant load removal were associated with a higher concentration of contaminants in the effluent, following the principle that the greater the cell density within an algal suspension, the faster the organic matter would be integrated into the metabolic processes of the microorganisms [72,73].

### 3.4. Efficiency of the System for Removal of Pathogens

Several studies attempted to define which factors directly lead to a reduction in fecal coliform content in algae-mediated WWT systems. Ansa et al. [74],Almasi and Pescod [75] reported the following as possible factors: concentration of contaminants and dissolved oxygen content in the effluent, occurrence of toxins released by microalgae, exposure to solar radiation, and temperature of the growth medium. Marchello et al. [76], meanwhile, ascribed the causes of a significant reduction in coliform content to pH changes in the growth medium due to photosynthesis and respiration processes. Moreover, Ahmad et al. [77] claimed a negative effect of competition for nutrients due to the increase in algal biomass density in the growth medium.

Since in the first experiments performed in Las Cruces with *G. sulphuraria* it was observed a complete reduction of the bacterial load within 4 days, it was subsequently decided to study the phenomenon with respect to the variation of different process variables, in order to achieve a systematic characterisation of the bacterial response to the operating conditions of the algal system [51,52,63]. The parameters selected for investigation were pH and temperature of the effluent, photo-oxidation, presence of algal biomass that could adsorb the bacterial load, synergistic effect between the above parameters.

About the pH, bactericidal activity in the algal system was found to be guaranteed by a low pH value in the growth medium, despite it being a condition not sufficient alone. In fact, according to literature, many pathogenic bacteria can resist low pH; in particular, Audia et al. [78] reported that enteropathogens including *Escherichia coli*, *Salmonella typhimurium*, and *Helicobacter pylori* could survive at very low pH of 2–3 by employing different acid resistant mechanisms. First data coming from Las Cruces experiments about monitoring the fecal bacterial community in POWER system relied on the study of the concentration of total *E. coli*, whose optimal pH for growing is in the range 5.8–8.0 [79]. As expected, tests conducted at pH 7.0 and pH 6.3 did not provide any satisfying reduction in the *E. coli* concentration after 8 h, despite the colony count at pH 6.3 being lower than the one reported for pH 7. Instead, in the test reactor maintained at pH 4 it was reported an *E. coli* colony count near-zero (no colonies were observed) [52]. Temperature was not a factor impacting the survival of pathogens, which were able to grow in their temperature range even in the presence of *Galdieria* and died when the temperature exceeded the optimum range. Again considering *E. coli* as a reference organism, it grew between 30 and 40 °C and degenerated above 40 °C [52]. This was in agreement with literature, as the optimum temperature range for *E. coli* growth is 20–40 °C [78,80], preferably 37 °C [81]. Regarding the possible attachment to algal biomass, no significant statistical difference in the number of *E. coli* was observed when comparing presence *vs* absence of algal biomass into the effluent. On the other hand, other factors that were proven to directly affect bacterial load reduction, especially when combined together, were sunlight and dissolved oxygen. The presence in the growth medium of exogenous and endogenous photo-sensitizers, as for example humic substances and as porphyrins (both being abundant in municipal wastewater), could induce the formation of reactive oxygen species that could cause cell damage to bacteria [82]. A further factor that could explain a higher toxicity in the *G. sulphuraria* algal system rather than the conventional activated sludge process might be due to the presence of trace metals such as Mg^2+^, Ca^2+^, Fe^3+^ and Cu^2+^ [13], which needed to be supplied in the photobioreactor at the start of each batch cycle for supporting algal growth. As a proof, when such metals were added to the effluent, vital functions of *E. coli* immediately ceased, neither *E. coli* showed regrowth in the subsequent 3 days of observation [52]. Finally, testing the synergetic effects of algal biomass, metabolites, temperature, and sunlight, it was observed an inactivation of native fecal coliform in the primary effluent also when pH level was 7, demonstrating that all the operating conditions of the power system could jointly ensure proper disinfection of the effluent [52,63]. Nevertheless, when the tests were undertaken in an acidic medium, performances were improved further, supporting the assumption that the lower was the pH, the faster was the pathogen inactivation [51].

Following these initial experiments, further sampling and studies were conducted to investigate the pathogenic aspects of the POWER process from a genomic perspective, including DNA sequencing [35,42,45]. Initially, Delanka-Pedige et al. [35] compared the changes in the bacterial community of wastewater treated with both conventional activated sludge and algal systems, showing that the process performed by *G. sulphuraria* was able to reduce the total coliform count by more than 7 logs, while the putative bacterial community in the sewage was almost completely reduced (to undetectable levels). Later, still comparing POWER system to the conventional wastewater treatment process, in [45] the objective was to study the evolution of the viral community in the wastewater, demonstrating not only that the algal process was able to reduce the viral load in a single step to levels that could only be reached after the chlorination phase in conventional WWTPs, but also that the viral community in the wastewater treated by *G. sulphuraria* was much less diverse (14 species versus approximately 250). Lastly, the most recently reported bacteriological study in the framework of the POWER process focused on the variation in the concentration of bacteriophages in the wastewater, as these organisms actively promote bacterial resistance to antibiotics through gene transfer [42]. Again, the *G. sulphuraria*-mediated effluent purification process proved to be more effective than conventional purification treatments, providing a significant reduction in phage-mediated transfer of 80% of antibiotic resistance genes.

### 3.5. Use of Hydrothermal Liquefaction to Maximise Energy Recovery and Biomass Production

Biomass of microalgae belonging to *Galdieria* genus is composed mainly of carbohydrates, followed by proteins and then lipids. Their proportion within the cell was described to vary with culture conditions. The study on *G. phlegrea* demonstrated the possibility of extracting an average lipid content of 17% in biomass grown in municipal wastewater [46]. Considering the low *Galdieria* lipids content, which makes their extraction unconvenient, hydrothermal liquefaction (HTL) rose as an alternative to produce biofuels from such biomass. Hydrothermal Liquefaction (HTL) is a recently emerged technology that allows to process wet biomass under moderate temperatures (150–350 °C) and pressures (15–20 MPa) [83]. During the process, the biochemical compounds (e.g., lipids, proteins and carbohydrates) constituting algal biomass are transformed in energy-dense bio-crude oil through complex chain reactions [84]. Such bio-crude can be fractioned into boiling fractions analogous to gasoline, diesel and/or fuel oil and used in combination with petroleum products or even refined [85,86]. Along with bio-crude oil, HTL process also generates solid and liquid fractions, respectively represented by biochar and an aqueous phase, which is rich in organic macronutrients (C, N, P). Since HTL process ensures multiple benefits from an energetic point of view, it sets as an opportunity to avoid the economic challenge posed by biomass drying step in conventional algal cultivation processes, which requires a major energy consumption to be properly performed [87].

While early HTL studies were aimed at maximizing bio-crude yield, the focus of experiments described in [40,41,43,44,59,60,61] was to assess beneficial reuse of the aqueous phase and evaluate the overall energy balance of the process. About the first aspect, many studies successfully attempted to demonstrate the possibility of boosting biomass productivity by diluting small concentrations of aqueous phase in the algal growth media [88,89]. The rationale behind these experimentations lied in the fact that aqueous phase was rich of sterile macronutrients; hence, its recycling into a PBR for microalgae cultivation purposes mediated by wastewater could compensate the stoichiometric imbalance (C:N:P ratio) between algal biomass and municipal effluent [59]. Since the aqueous phase was usually recycled in diluted form, the residual fractions might be potentially repurposed for economically feasible fertilizer production, or to improve the cultivation of more microalgae in other PBRs with different water supply.

Results obtained from the tests conducted in Las Cruces with the *G. sulphuraria*-mediated process showed a significant increase in biomass growth (+20%) compared to control medium (33.92–30.23 g_biomass_ g _N removed_^−1^), while ammonium and phosphate removal rates remained stable in the ranges 95.2–99.7% and 96.2–99%, respectively. The removal rates of NH_3_-N ranged between 4.71–4.97 mg L^−1^ d^−1^, while the removal rates of phosphate ranged between 1.47–1.68 mg L^−1^ d^−1^. Results showed that ammonium and phosphate removal in this system complied with the discharge standards mandated by the local regulatory agencies.

### 3.6. Performance of the System in a Simulated Continuous Process

The final step in advancing the POWER system towards continuous operation was evaluating its performance in fed-batch mode [41,63]. The semi-continuous mode was simulated through cycles. Each cycle started supplying fresh wastewater to the PBR, and it ended when the reactor contents reached the discharge standard for BOD_5_, ammoniacal-nitrogen (N), and phosphates (P). At the end of each cycle, before starting a new one, biomass was allowed to settle for sampling reasons and more than half of the supernatant was replaced again [53].

In a process designed in this way, Tchinda et al. [63] reported that algal biomass accumulated and continued to grow at nearly the same rate throughout all the fed-batch cycles, without showing any lag phase and reaching a maximum OD at 750 nm of 1.3. The intuition of accumulating biomass during the fed-batch cycles (instead of harvesting at the end of every batch operation), enabled higher removal rates of the pollutant load. In particular, starting from an average concentration of ammoniacal-nitrogen in the primary effluent of about 33.9 ± 6.1 mg L^−1^, it was possible to comply with the local discharge standard of 10 mg L^−1^ for N in about 2 days [63]. Phosphate and BOD_5_ both followed the same reduction trend of ammonium during the same time interval. Concerning removal rates, Tchinda et al. [63] reported ~6.00 ± 1.00 mg L^−1^ d^−1^ and ~1.40 ± 0.58 mg L^−1^ d^−1^ for the removal of ammonium and phosphate—respectively—from municipal wastewater, which were values higher than what previously obtained in laboratory batch studies (Section 3.3).

## 4. Future Prospects

The previous sections described the main outcomes of the research on the use of microalgae of *Galdieria* genus for purposes of wastewater treatment. Until now, research efforts led to the design and assessment of an effective system for the removal of contaminants from urban wastewater and the simultaneous production of algal biomass. This biomass could eventually be used for the sustainable production of by-products and energy, thus meeting the demand for circular economies and biorefinery policies. All the expertise built up since 2014 on the use of microalgae of the genus *Galdieria* for the purification of urban wastewater paves to the exploration of new pathways towards efficient cultivation processes and the implementation of increasingly sustainable sewage treatments, in order to address current environmental and climate issues in a tangible way.

A first challenge is the development of photobioreactors that optimize the use of the land surface, while obtaining satisfactory biomass yields and reducing the energy demand required to cover the most critical stages of the process (harvesting and drying of biomass). Hence, upcoming experiments should move towards the development of less energivorous cultivation systems with attached biomass, for which *Galdieria* already showed great affinity, successfully achieving (in experiments not involving urban wastewater) higher biomass yields than growth in liquid phase [38].

Given the extraordinary capacity of *Galdieria* to remove pollutants from municipal wastewater, a further challenge for research could be the possibility of obtaining potable water from different sources of liquid waste, including not only sewage [50] but, for example, also wastewater from vegetables-washing processes in the food industry [56]. In this scenario, the implementation of coupling strategies of genetic optimization of nutrient uptake to boost the contaminant removal ability, as suggested by Perin et al. [9], could emerge as a winning strategy.

The present work aimed at shifting the attention to the use of extremophilic organisms for industrial applications which do not usually employ them, while summarizing the multiple advantages of their use. The experimentation of processes that can combine the exploitation of biodiversity with the achievement of sustainability goals is an approach that should be pursued in the fight against climate change.

## Figures and Tables

**Table 1:** Scientific articles provided by Scopus platform and associated to the words “*Galdieria*” and “wastewater”. Articles included into the study (“eligible”) are the ones involving municipal wastewater as effluent.

Reference	Effluent Tipology	Strain	Highlights	Eligible
[37]	Municipal wastewater	*G. sulphuraria* CCMEE 5587.1	Prediction of the operational cycle time that is needed to comply with the discharge levels imposed by current legislation for ammonium, phosphate and BOD_5_ removal, in a fed-batch cultivation system.	yes
[38]	*Galdieria* medium	*G. sulphuraria* ACUF 064	Comparing the growth of *G. sulphuraria* in 5 different cultivation systems, four being in liquid phase and one on the innovatine Twin Layers photobioreactor.	no
[39]	f/2 growth medium with 2% ocean salts	*G. sulphuraria* CCMEE 5587.1	Characterization of the lipid profile of pyrolysis oil derived from the hydrothermal liquefaction of *G. sulphuraria*, compared with the bio-crude extracted from *N. salina*.	no
		*N. salina* CCMP1776		
[40]	Municipal wastewater	*G. sulphuraria* CCMEE 5587.1	Comparing overall performances of hydrothermal liquefaction processes performed continuously (4 h) and in batch systems.	yes
		*G. sulphuraria* polyculture		
[41]	Municipal wastewater	*G. sulphuraria* CCMEE 5587.1	Comparing overall performances of hydrothermal liquefaction processes performed continuously and usign two different strains of *G. sulphuraria*, to simulate seasonality and different response to the surrounding environment.	yes
		*G. sulphuraria* SOOS		
[42]	Municipal wastewater	*G. sulphuraria* CCMEE 5587.1	Comparing removal of antibiotic resistant bacteria and antibiotic resistant genes in two different systems: algal-based system employing *G. sulphuraria* and conventional activated sludge process.	yes
[43]	Municipal wastewater	*G. sulphuraria* CCMEE 5587.1	Evaluate the addition of different alcohols to the biomass, in order to improve the performances of hydrothermal liquefaction processes.	yes
[44]	Municipal wastewater	*G. sulphuraria* CCMEE 5587.1	Exploring performance and changes in bio-crude oil chemistry of a hydrothermal liquefaction process in which crude glycerol was added to algal biomass, and a following step of catalytic upgrading using Pt/C was performed.	yes
[35]	Municipal wastewater	*G. sulphuraria* CCMEE 5587.1	Monitoring and comparing the total content of pathogens in the wastewater (from a genetic sequencing point of view) treated with: *G. sulphuraria* algal-system, conventional activated sludge process.	yes
[45]	Municipal wastewater	*G. sulphuraria* CCMEE 5587.1	Monitoring the reduction of somatic and F-specific coliphages in the wastewater as indicators of enteric viruses.	yes
[46]	Municipal wastewater	*G. phlegrea* ACUF 784.3	Testing for the first time the growth of *G. phlegrea* in urban wastewater under laboratory conditions and in batch mode. Metabolic pathways of Carbon and Nitrogen from the growth medium to the biomass were evaluated using isotopic analysis.	yes
[47]	Municipal wastewater	*G. sulphuraria* CCMEE 5587.1	Comparing performances of *G. sulphuraria* in terms of biomass growth and contaminant removal in various growth media, prepared according to different criteria.	yes
[13]	Municipal wastewater	*G. sulphuraria* CCMEE 5587.1	Comparing the results obtained in terms of biomass growth, N, P and BOD_5_ removal, when scaling-up from laboratory to field conditions.	yes
[48]	Sulfuric acidic hot springs	*G. sulphuraria* 074G	Monitoring the growth of microalgae in an acidic extremophilic environment (hot springs).	no
		*Pseudochlorella* sp. YKT1		
[49]	Solutions of uranium, vanadium, and titanium	*G. sulphuraria* SBU-SH1	Evaluating biosorption capacity of heavy metal ions by a novel strain of *G. sulphuraria* identified and presented for the first time.	no
[50]	Municipal wastewater	*G. sulphuraria* CCMEE 5587.1	Investigating the technical feasibility of recovering potable-quality water from a system for wastewater treatment mediated by *G. sulphuraria*.	yes
[22]	Modified Allen’s Medium	*G. sulphuraria* 074W	Evaluating the biosorption capacity of precious metals such as gold, platinum, and palladium by *G. sulphuraria*, and the subsequent elution capacity of such heavy metals.	no
	Metal wastewater solutions (Au^3+^, Pd^2+^, Pt^4+^)			
[51]	Municipal wastewater	*G. sulphuraria* CCMEE 5587.1	Mathematical modelling of the correlation between coliform content in wastewater and the following parameters: temperature, pH, light, combination of parameters.	yes
[52]	Municipal vastewater	*G. sulphuraria* CCMEE 5587.1	Practical testing of the correlation between coliform content in wastewater and the following parameters: algal metabolites, pH, temperature, algal biomass attachment, sunlight and algal biomass, sunlight and dissolved oxygen, synergistic effect.	yes
[53]	Municipal wastewater	*G. sulphuraria* CCMEE 5587.1	Testing the performances of the single-step system for cultivation of *G. sulphuraria* and wastewater treatment using a fed-batch process with 3-day cycles. The results concern the ability to remove N, P, BOD and pathogen load.	yes
[54]	Produced water	*G. sulphuraria* CCMEE 5587.1 *C. vulgaris* UTEX 395	Comparing the growth performance of two algal strains with a culture medium composed of Produced Water deriving from oil extraction activities.	no
[55]	Second cheese whey	*G. sulphuraria* SAG 107.79	Evaluating the possibility to cultivate *G. sulphuraria* with Second Cheese Whey and the performances of the process.	no
[56]	Wastewater from fruit-salad production	*G. sulphuraria* SAG 21.92	Monitoring algal growth and sugar consumption in wastewater from fruit-salad production.	no
[57]	Municipal wastewater	*G. sulphuraria* CCMEE 5587.1	Studying the feasibility to use urban wastewater as a growth medium for *G. sulphuraria* and the simultaneous reduction of the main effluent contaminants.	yes
[58]	Municipal wastewater	*G. sulphuraria* CCMEE 5587.1 and CCMEE 5572	Comparing the performances of two dirreferent *G. sulphuraria* strains in terms of growth and removal of contaminants. The metabolic response of the two strains was evaluated also in relation to temperature and growth media.	yes
[59]	Municipal wastewater	*G. sulphuraria* CCMEE 5587.1	Testing the effects on biomass growth and contaminants removal of diluting into the wastewater the aqueous products of hydrothermal liquefaction process.	yes
	Acqueous phase from HTL process			
[60]	Municipal wastewater	*G. sulphuraria* CCMEE 5587.1	Characterizing NH_3_, total N, P and carbohydrates content in the aqueous phase of hydrothermal liquefaction process as a function of the operating temperature. A characterization of the overall energy yield of the process was also made, compared to the performance of other strains reported in the literature.	yes
	Acqueous phase from HTL process			
[61]	Municipal wastewater	*G. sulphuraria* CCMEE 5587.1	First presentation of the system denominated “POWER” for single step wastewater treatment mediated by *G. sulphuraria*, and coupled with hydrothermal liquefaction for the production of high-value energy compounds.	yes
[62]	Allen’s standard cyanidium medium with H_2_PtCl_6_	*G. sulphuraria* UTEX 2919	Testing the ability of *G. sulphuraria* to bio-remove negative charged metal complex PtCl_6_^2−^ from synthetic wastewater.	no
[63]	Municipal wastewater	*G. sulphuraria* CCMEE 5587.1	Testing growth and contaminants removal performances of *G. sulphuraria* in a cultivation system where it was simulated a fed-batch process, by replenishing a fraction of the wastewater with fresh effluent every 3 days.	yes
[64]	Artificial growth media. Paper based on data from literature, not real experiments.	*G. sulphuraria* (strain not specified)	Theoretical modelling and evaluation of a combined cultivation process with gas exchange between phototrophic and heterotrophic growth conditions.	no

## Data Availability

No new data were created or analyzed in this study. Data sharing is not applicable to this article.
